# Determination of amino acids in human biological fluids by high-performance liquid chromatography: critical review

**DOI:** 10.1007/s00726-021-03002-x

**Published:** 2021-05-24

**Authors:** Grażyna Gałęzowska, Joanna Ratajczyk, Lidia Wolska

**Affiliations:** Department of Environmental Toxicology, Faculty of Health Sciences, Medical University of Gdansk, Debowa Str. 23A, 80-204 Gdańsk, Poland

**Keywords:** Amino acids, HPLC, Sample preparation, Urine, Blood, Cerebrospinal fluid

## Abstract

The quantitation and qualification of amino acids are most commonly used in clinical and epidemiological studies, and provide an excellent way of monitoring compounds in human fluids which have not been monitored previously, to prevent some diseases. Because of this, it is not surprising that scientific interest in evaluating these compounds has resurfaced in recent years and has precipitated the development of a multitude of new analytical techniques. This review considers recent developments in HPLC analytics on the basis of publications from the last few years. It helps to update and systematize knowledge in this area. Particular attention is paid to the progress of analytical methods, pointing out the advantages and drawbacks of the various techniques used for the preparation, separation and determination of amino acids. Depending on the type of sample, the preparation conditions for HPLC analysis change. For this reason, the review has focused on three types of samples, namely urine, blood and cerebrospinal fluid. Despite time-consuming sample preparation before HPLC analysis, an additional derivatization technique should be used, depending on the detection technique used. There are proposals for columns that are specially modified for amino acid separation without derivatization, but the limit of detection of the substance is less beneficial. In view of the fact that amino acid analyses have been performed for years and new solutions may generate increased costs, it may turn out that older proposals are much more advantageous.

## Introduction

Amino acids (AAs) are organic compounds, derivatives of hydrocarbons, containing an amino group (-NH_2_) and carboxyl group (-COOH). Their analysis using modern analytical methods has generated great interest in recent years. It is caused by the fact that AAs are a part of proteins and peptides, important building materials and the starting materials for the biosynthesis of certain hormones. AAs are the building blocks of proteins and can serve as a source of energy. Apart from their nutrition function and gene expression regulation, they play an extremely important role and essential function in the life processes of organisms.

AA profile is determined in human biological and environmental samples such as plants (Qureshi et al. 2013a, b) well air, urban dust (Buiarelli et al. 2013) or bioaerosols (Mandalakis et al. 2011). A profile in a biological sample is used for an early diagnosis of various diseases. They are the precursors of many biomarkers, for example tyrosine and proline for 3-nitrotyrosine and trans-hydroxyproline, respectively. Their presence in the exhaled air can be indicative of nitrosative stress in the lungs (Conventz et al. 2007; Göen et al. 2005). In the plasma of autistic children, there are significantly lower levels of AAs such as leucine, isoleucine, phenylalanine, methionine and cysteine than in control samples (ElBaz et al. 2014). Similarly, in the case of insulin resistance, some disorders of AA profile were recognized (Harder et al. 2011). The levels of phenylalanine, leucine, valine, citrulline and methionine have been determined for the diagnosis of phenylketonuria, maple syrup urine disease, citrullinemia and homocystinuria, respectively (Chen et al. 2014; Qu et al. 2001; Schulze et al. 2003). Inborn error of metabolism can be determined on the basis of the amino acid profile (Held et al. 2011; Roux et al. 2011). Besides, enzymopathies (e.g. phenylketonuria and maple syrup urine disease) and disorders of AA transport (e.g. cystinuria) labeled AA profile can help in assessing the nutritional status of humans, the dietary compliance, renal function, tissue damage and neuropathy problems (Le et al. 2014; Li et al. 2011a, b). An interesting use of the AA profile is its correlation with pain. AAs also appear to transmit pain signals and may be involved in this syndrome (Larson et al. 2000). Until recently, such health problems were attributed only to the composition of L-amino acids. Recent studies have shown that d-amino acids (D-AAs) are present in organisms at a higher level and to a greater extent than previously considered. D-AAs meet certain biological functions in the body (Müller et al. 2014), and are considered as new bioactive compounds and biomarkers (Hammase et al. 2009). Sato et al. (2021) have provided research showing that L-type amino acid transporter 1 is associated with chemoresistance in breast cancer via the promotion of amino acid metabolism. Moreover, in case of nonalcoholic fatty liver disease, the alterations in plasma concentrations of amino acids and their derivatives were observed (Tricò et al. 2021). Yamakado et al. (2017) identified increased plasma levels of 12 amino acids and lower levels of 3 amino acids compared with samples of healthy people.

Therefore, the analysis of AA profiles is an important tool for the study of metabolomics regulation and dysregulation. Their identification and separation are difficult due to the high polarity of these compounds, their low volatility and absence of strong chromophoric groups. It should be noted that the use of MS has already had a significant application in some clinical areas for example to detect inborn errors of metabolism (Klinke et al. 2020). This group of diseases with variable incidences are caused by disrupting enzyme activities in metabolic pathways. Metabolomics included AA analysis may allow to widen the scope of diagnostics.

Historically, the oldest technique (but still widely used for the determination of AAs), is ion chromatography, used for the first time by S. Moore and W.H. Stein in 1948 (Moore and Stein 1948). In 1958, together with Spackman, they published the description of the automatic AA analyzer for quantitative and qualitative determination of amino acid content of the protein based on ion exchange column chromatography after post-column derivatization with ninhydrin. For this work, in 1972 they were awarded the Nobel Prize. Unfortunately, chromatographic separation of all AAs takes up from 60 to 150 min, which severely limits sample throughput. Furthermore, these instruments are expensive and if the sample load is not large enough, it is not cost-effective considering the high price (Johnson 2011). In spite of many unfavorable factors, these analyzers are still used because they are fully automated. High-performance chromatographic methods are becoming increasingly important in the determination of AAs. The reasons for the implementation of new applications of AA analysis are shorter analysis time, lower costs and higher sensitivity.

Novel procedures include reversed phase or ion pair liquid chromatography and mass spectrometer detectors. This is often associated with sample preparation for isolating AAs from matrix (Calderón-Santiago et al. 2012; Fekkes, 1996). Frequently, the pre- or post-column derivatization reactions are used (Kőrös et al. 2007; Li et al. 2011a, b; Sharma et al. 2014; Ubhi et al. 2013; Waldhier et al. 2010) for each analysis to increase hydrophobicity or structural stability of metabolomics compounds. Analysis by liquid chromatography with ultraviolet (Qu et al. 2001), fluorescence (Kőrös et al. 2007; Li et al. 2011a, b; Sharma et al. 2014) and mass spectrometry (Johnson, 2011; Kaspar et al. 2009; Ubhi et al. 2013) may require the introduction of the chromophore, fluorophore or ionization group.

The key problems faced in the metabolomic analysis include:

- choosing a technique that allows the isolation of compounds with the highest possible recovery,
- choosing one technique allowing for separation and detection of as many compounds as possible in biological samples, but with high sensitivity and reliable detection.

The purpose of this review was to evaluate the new solutions for sample pretreatment and liquid chromatography analysis of AAs in physiological fluids such as urine, cerebrospinal fluid or blood. These data could help in the updating and compilation of analytical techniques that have been so far used in the analysis of AAs. Particular attention was paid to identify the advantages and disadvantages of different techniques used for the separation and determination of AAs.

## Sample preparation

Sample preparation is often a critical step in the analysis of AAs. The number of operations during sample preparation should be minimized to reduce sources of errors and analysis time. New green sample preparation methods are becoming more common because they are more environmentally friendly due to no use of toxic reactants and solvents. Optimal sample preparation should reduce analysis time, sources of error, enhance sensitivity and enable unequivocal identification and quantification of the AAs. However, to reduce matrix interferences (especially in biological samples) some sample preparation steps are necessary.

The choice of sample preparation method for biological fluids during the determination of amino acids depends mainly on the matrix. Most of the existing studies dealing with AA analysis have focused mainly on blood, urine and cerebrospinal fluid.

Blood may be processed as serum or plasma. Plasma, which constitutes 55% of blood fluid, is mostly water (92% by volume), and contains dissipated proteins, glucose, mineral ions, hormones, carbon dioxide, and blood cells themselves. Serum is also the liquid portion of coagulated blood, not including clotting factor (anticoagulant), which is added to blood during plasma preparation (Jambunathan and Galande 2014). Although serum and plasma are usually considered to have similar compositions and properties, AA concentrations in plasma and serum differ. The incubation of blood specimens affects the analyte peak areas in serum less than those in plasma. Consequently, serum is recommended as the chosen sample in metabolomic studies (Liu et al. 2010).

In AA studies, cerebrospinal fluid (CSF) can also be used as a sample. It is produced in the choroid plexuses of the ventricles of the brain (Killer et al. 2006; Reiber 2001). The composition of CSF is dependent on the filtration process (blood–brain barrier). The CSF contains a variety of proteins, of which 85% are derived from the blood, whereas approximately 15% are synthesized primarily in the brain (Di Chirio 1964; Felgenhauer 1974).

In contrast to blood or CSF, urine samples are exceptionally convenient for researches due to the availability of a large amount of this biological material and sampling simplicity. Additionally, in urine proteins (albumin and globulins) are presented at relatively low concentrations. The dynamic range of protein concentrations in urine is smaller than in serum or CSF (Pastushkova et al. 2012). Urine is composed of water (91–96%) but also mineral salts, metabolites and other substances (Rose et al. 2015).

Consequently, to relate the content of AA compounds in the final extract to their concentration in body fluids, using proper sample storage, cleaning up to remove salts or proteins or derivatization are needed (see Table 1).

**Table 1:** Summary of data on the storage and sample preparation parameters of human fluids containing amino acids

Type of sample	Storage/preservation	Deproteinization	Derivatization	AA isolation/cleanup/desalination	References
Blood	Lithium heparin vacutainer tubes and centrifugation at 2003×*g*. for 15 min, decantation of plasma and storage	–	–	–	ElBaz et al. (2014)
	Collection in K_2_EDTA, centrifugation for 10 min at 2000×*g*, stored at − 80 °C	–	–	–	Lewis et al. (2008)
	Incubation for 30 min at room temperature, centrifugation at 2000×*g*. for 15 min at 4 °C, storage at − 80 °C until analysis	Addition of methanol (1:6, v/v), immersion in an ice bath, shaking for 1 min, centrifugation at 13,800×*g*. for 5 min at 6 °C, dilution of upper phase with methanol (2:5, v/v)	–	SPE automated, ion exchange SPE column, conditioning: acetonitrile and 50% acetonitrile with 1% formic acid, sample load: in 90%acetonitrille with 1% formic acid, washing: 70% acetonitrile, elution: 90% acetonitrile containing 5% ammonium hydroxide	Calderón-Santiago et al. (2012)
		Addition to serum of perchloric acid (0.624 M), centrifugation for 10 min at 10,000×*g* at 4 °C	–	–	Li et al. (2011a, b)
		Addition of 0.1 N hydrochloric acid in methanol, shaking 10 min, stored for 2 h at − 18 °C, centrifugation for 10 min at 2300×*g*. at 4 °C	Derivatization of AAs to AA butyl esters using butanolic hydrochloric acid, completely drying of supernatant under nitrogen, addition of hydrogen chloride (3 M in 1-butanol), shaking, 1000×*g*. for 15 min at 60 °C, completely drying of supernatant under nitrogen, solution at water: methanol (8:2, v/v) and 0.1% formic acid	–	Harder et al. (2011)
		Drying solution of methanol, addition of acetonitrile (1:10, v/v)	Mixing of plasma acetonitrile and labeling reagents in acetonitrile with 1% trifluoroacetic acid, Heating at 65 °C for 30 min, Quenching with hydroxylamine (12% in water), Dilution with water: acetonitrile: formic acid ( 50:50:0.1, v/v/v)	–	Johnson, (2011)
		Addition of methanol, After 20 min, centrifugation, evaporation of supernatant	Resolvatation in HCl/butanol, incubation at 65 °C for 15 min, evaporation to dryness, reconstitution in solvent of acetonitrile:water:formic acid (50:50:0.025, v/v/v)	–	Schulze et al. (2003)
				C18 POLAR RP SPE column, conditioning: ammonium acetate buffer (pH 4.35) and methanol (60/40, v/v) and ammonium acetate buffer (pH 4.35), sample load: with ammonium acetate, washing: ammonium acetate buffer (pH 4.35), elution ammonium acetate buffer (pH 4.35) and methanol (6:4, v/v)	Göen et al. (2005)
	After 1 h at room temperature, centrifugation at 2500×*g* for 15 min, stored at − 70 °C until analysis	Addition of acetonitrile (1:10, v/v), vortex mixing for 0.5 min, standing for 15 min, centrifugation at 15,000×*g* for 15 min	Mixing of extracted serum, acetonitrile, borate buffer and labeling reagent (25:100:60:25, v/v/v/v), standing at room temperature for about 6.3 min, addition of 50% acetic acid	–	Li et al. (2011a, b)
	Collection in heparinized vacutainers, centrifugation for 7 min at 1200 rpm. at 4 °C	Addition of 0.1 M HCl (1:5, v/v), filtration	Mixing of filtrate aliquot with phenylisothiocyanate, drying under vacuum, resolution in sample diluent		Svasti et al. (2001); Vatanavicharn et al. (2012)
	Incubation for 30 min at room temperature, centrifugation at 2500×*g* for 15 min	Addition of methanol (1:6, v/v), 4 times, centrifugation at 13,000×*g* for 15 min at 4 °C, dilution of upper phase with methanol (2:5, v/v)	Mixture of pretreated serum samples, aqueous–organic reaction solution and di-isopropyl phosphite, immersion in ice-water bath, mixing periodically on vortex for 30 min Evaporation under gentle flow of nitrogen at room temperature, redissolution with 0.1% formic acid	Sep-Pak1 Vac C18 SPE column, conditioning: methanol and 0.1% formic acid, sample load: in 0.1% formic acid, washing: 0.1% formic acid, elution: 0.1% formic acid–methanol (v/v, 1:9)	Chen et al. (2014)
	Incubation on ice, containing lithium-heparin, centrifugation immediately for 10 min at 4000 rpm at 4 °C, Incubation on ice	Addition of trichloroacetic acid (10:1, v/v), mixing on vortex, frozen in liquid nitrogen, storage at − 80 °C until analysis	Addition of derivatization agent (4: 50, v/v), mixing on vortex for 10 min, reaction for 1 min, addition of organic extraction solution (propylchloroformate in chloroform), mixing on vortex for 0.3 min and standing for 1 min, transfer organic solution, evaporation under nitrogen gas, dissolution with mixture of methanol/water (62:38, v/v)	Commercial AA analysis kit, ion exchange SPE column,	Meesters et al. (2009)
Plasma	Stored at − 4 °C	–	–	Transfer samples to PEME sample vial, dipping of fiber in organic solvent for 5 s, Wiping away the excess of organic solvents, introduction of solution of acceptor phase into the lumen of the hollow fiber, introduction of cathode into the fiber lumen, connection of electrode to the power supply, mixing with stirring rate of 1250 rpm for 20 min while the pulse frequency was 3 min^− 1^, collection of extract by microsyringe	
	–-	Addition of solution methanol: acetonitrile to plasma sample (6:6:2, v/v/v), centrifugation for 40 min at 20,000×*g*, evaporation to dryness	Adjustment pH with borate buffer solution, mixing with derivatization reagent, incubation for 5 min at 60 °C, cooling the reaction mixture in ice water, addition of HCl solution to reaction mixture	–	Song et al. (2013)
Serum, plasma	Collection with EDTA, stored at − 80 °C	Incubation on ice, addition of ice cold methanol, centrifugation for 15 min at 15000 rpm at 4 °C, evaporation of supernatant, resolution in water	Mixing of methanolic solution of ortho-phthalaldehyde with chiral thiol isobutyryl-l-cysteine (in borate buffer), adjustment of pH to 9 with sodium hydroxide, incubation for 2 min	–	Müller et al. (2014)
Urine		Addition of 10% sulfosalicylic acid (1:4, v/v), Mixing on vortex, Centrifugation for 3 min at 14,000×*g*	–	–	Held et al. (2011)
	Adding of boric acid, stored in liquid nitrogen or at − 20 °C until analyses	Addition of 10% sulfosalicylic acid (4:1, v/v), mixing for 0.5 min, centrifugation for 5 min at 700×*g*	Mixing of supernatant with labeling buffer (1:4 v/v), mixing of diluted supernatant with itraq® reagent and solution (2: 1: 23, v/v/v), incubation at room temperature for 30 min, addition of 1.2% hydroxylamine, overnight evaporation, reconstituting with iTRAQ® reagent	–	Kaspar et al. (2009)
	Stored at − 80 °C	Incubation of ice, centrifugation for 15 min at 15000 rpm at 4 °C	Mixing of methanolic solution of ortho-phthalaldehyde with chiral thiol isobutyryl-l-cysteine (in borate buffer), adjustment pH to 9 with sodium hydroxide, incubation for 2 min	–	Müller et al. (2014)
	––	–	Commercial kit, chloroformate derivatization	Commercial kit, spe, liquid/liquid extraction, drying under nitrogen, reconstituted in solution of ammonium formate in water:ammonium formate in methanol solution.(1:2, v/v)	Ubhi et al. (2013)
Plasm, urine, and cerebrospinal fluid		Addition of 6% sulfosalicylic acid (1:1, v/v), incubation at room temperature for 5 min, centrifugation for 5 min at 13,000 rpm dilution with 2 mM tridecafluoroheptanoic acid (1:80, v/v)	–	–	Le et al. (2014)
	Blood: collection in heparinized and EDTA–fluoride vacutainers; centrifugation for 10 min at 35,009×*g*	Reduction of disulphide bonds and to release bound Cys from proteins, Mixing on vortex for 0.5 min, addition of 10% sulfosalicylic acid, Mixing on vortex for 2 min, centrifugation for 10 min at 25000 rpm at 4 °C	Adjustment pH to 8.8 with flour borate buffer, mixing with derivatization reagent (in acetonitrile), incubation for 210 min at 55 °C	–	Sharma et al. (2014)
Cerebrospinal fluid	Stored at − 80 °C	–	–	Dilution (1:4, v/v) of sample with a mixture of acetonitrile/methanol/water (65:25:10, v/v/v),	Voehringer et al. (2013)
	Stored at − 80 °C	–	OPA/MCE derivatization, incubation 3 min	–	Voehringer et al. (2013)

The first stage of sampling and storage may significantly influence the obtained results. A sample of venous blood should be collected early in the morning after an overnight fast. The serum fraction should be isolated up to 2 h after collection of the blood sample (Calderón-Santiago et al. 2012). The usage of clotting factors can affect the AA concentration. This is related to bonding AAs by anticoagulant (Liu et al. 2010). For coagulation without clotting factors, the sample should be incubated at room temperature. The samples should be stored minimum at 4 °C before further preparation. At higher temperatures, compound hemolysis can occur. This process causes erroneous results for aspartic acid, glutamic acid and taurine. Before freezing, the sample should be centrifuged. When samples are subjected to centrifugation 1500×*g* or lower, 5–15 min, this leads to an erroneously high concentration of taurine. To remove blood platelets, the recommended centrifugal speed should be higher than 2500×*g* (Fekkes 1996).

The sampling time is not important for CSF but samples should be kept frozen until assayed. To denature any enzymes that might alter the AA concentration, an acid (e.g. perchloric acid) can be added to the sample (Baker et al. 1993).

In case of urine sampling, collection should be done at defined times of the day or even better a 24 h collection (independent of circadian rhythms) (Fekkes 1996). Unfortunately, the volume of urine can be altered by a wide variety of factors such as water intake, perspiration and renal conditions. If sampling over 24 h has not been completed, an internal standard against the creatinine value can be used. The level of creatinine in urine decreases with increasing urine volumes (Rose et al. 2015). The urine samples, like serum or CSF, should be stored minimum at 4 °C.

The deproteinization step should be done immediately after centrifugation, otherwise, the temperature of sample collection should be decreased at minimum to -18 °C, to prevent degradation of proteins (Fekkes 1996). Protein precipitation is needed to eliminate negatively charged salts and other sources of ion suppression (e.g. phospholipids (Harder et al. 2011)).

Several methods for deproteinization of biological samples are known. The most commonly used are precipitation with acids and an organic solvent. Also, ultrafiltration and ultracentrifugation may be used. The last two do not appear to have been widely adopted because proteins are not removed completely and relatively expensive equipment is necessary (Fekkes 1996). Organic and inorganic substances can be used as precipitants, for example, methanol, acetonitrile, perchloric acid, hydrochloric acid, trichloroacetic acid, sulfosalicylic acid. The ratio between the volume of samples and the precipitation pattern is important. This should be determined experimentally, as it depends on the type of sample. After the addition of the precipitant, the samples should be mixed in all volumes. Until centrifugation, immersion in an ice bath can be used (Calderón-Santiago et al. 2012; Harder et al. 2011; Müller et al. 2014). For better separation, tubes with membranes can be used during centrifugation. More detailed information on precipitation can be found in the review by Fekkes (1996).

An alternative to deproteinization can be the extraction of AAs. These compounds are zwitterion compounds and they are highly hydrophilic species. This makes their extraction into organic solvents rather difficult. It should be done in the range of pKa (pH about 6) at which their net charge is neutral (Calderón-Santiago et al. 2012). Therefore, researchers are looking for new rapid and simple approach techniques, for example, electromembrane extraction of AAs from biological fluids such as blood and urine samples (Strieglerová et al. 2011).

Unfortunately, the sample preparation steps described above are not sufficient to measure AAs in the biological matrix. To determine AAs with low detection limit, time-consuming derivatization steps are often required. The derivatization methods have been extensively used to improve accuracy, reproducibility, and sensitivity. The use of derivatizing reagents for AA determination has undergone and continues to undergo major development over the course of many years (Fekkes 1996).

Derivatization enables the use of a specific type of detector and a change of hydrophobicity or structures of substances. This leads to the separation of not only AAs but also their enantiomers.

(*S*)-Naproxen–benzotriazole (Bhushan and Nagar 2013), 1-fluoro-2,4-di-nitrophenyl-5-L-alanine amide (Marfey’s reagent) (Bhushan and Nagar 2013) or -chloro-4-nitrobenzoxadiazole and 4-fluoro-7-nitro-2,1,3-benzoxadiazole (ElBashir et al. 2011) can be used as chiral derivatizing reagents, which allows separation and analysis of enantiomers. However, the use of most derivatization reagents is intended for a specific type of detector. Derivatization with phenylisothiocyanate allows the use of an ultraviolet–visible spectrophotometric (UV–Vis) detector (Svasti et al. 2001; Vatanavicharn et al. 2012).

In most studies (Li et al. 2011a, b; Müller et al. 2014; Sharma et al. 2014; Song et al. 2013; Voehringer et al. 2013), the identification and quantitation of AAs in biological samples was performed by high-performance liquid chromatography (HPLC) analysis with fluorescence detection (FLD), after derivatization of compounds with o-phthalaldehyde/mercaptoethanol (OPA) and 2-mercaptoethanol (MCE). It should be noted that besides derivatization of primary AAs, amino esters, amino alcohols, alkyl-aryl amines, heterocyclic amines can be applied. To use spectrometry detectors, substances like butanolic hydrochloric acid (Harder et al. 2011; Schulze et al. 2003) but also labeling reagents such as iTRAQ® (Held et al. 2011) can be used as derivatization reagents. The use of labeling reagents is criticized (Kaspar et al. 2009). It is considered that the cost of substances is high at a relatively low sensitivity of the spectrometer and the precision of some AA analyses is low.

All of the existing derivatization methods present various analytical problems: derivative instability, reagent interference, long preparation times, and inability to derivatize the secondary amino groups. The derivatization should be performed directly in the supernatant of the deproteinized samples, resulting in the quantitative recovery of AAa. Unfortunately, due to the incomplete removal of proteins, the substances can clog the chromatographic column (Johnson, 2011). To remove the residual salts generated in the reaction, desalination should be performed (Chen et al. 2014). To provide the optimum efficiency and selectivity of derivatization, the pH of the reaction is important. For that reason buffers are commonly used.

## HPLC analysis

HPLC has become one of the priority methods of choice for analyzing AAs. This is related to its high-throughput feature and ability to quantify various forms of compounds at low levels in biological fluids. Unfortunately, HPLC requires sample preparation for isolation or concentration of compounds. The sample volumes typically used for HPLC analysis are 20–50µL, while 200µL are required for the AA analyzer. A drawback of the usage of AA analyzer is the runtime of 130 min. In contrast, total runtime for an instrumental technique like HPLC could be a few minutes (Derezinski et al. 2017; Harder et al. 2011). HPLC is a powerful tool that enables the separation of complex mixtures into individual AAs. Compared to other analysis methods, it is advantageous in terms of shorter analysis times, greater resolution, and higher sensitivity. Additionally, HPLC is a highly sensitive and reproducible analytical technique. Over the years, HPLC has been combined with numerous detection methods and has steadily experienced an increasing number of new specific and selective stationary phases (see Table 2), which have enhanced its sensitivity and specificity for the determination of AAs.

**Table 2:** Summary of HPLC parameters of human fluids containing amino acids

Type of sample	AA	Column	Mobile phase	Flow [mL/min]	Detection	Calibration	References
Blood	Ala, Arg, Asn, Cit, Gln, Gly, Ile, Leu, Met, Orn, Phe, Tyr, Val	–	Acetonitrile: water: formic acid (50:50:0.025, v/v/v)	0.04	ESI MS/MS	–	Schulze et al. (2003)
	35 AA included: Ala, Arg, Asn, Asp, Cit, Cys, GABA, Gln, Glu, Gly, His, Ile, Leu, Lys, Met, Orn, Phe, Pro, Ser, Tau, Thr, Trp, Tyr, Val	RP column (300 × 3.9 mm, 46˚C)	A: sodium acetate buffer (pH 6.5):acetonitrile (97.5:2.5, v/v) B: acetonitrile:methanol:water (45:15:40, v/v/v) gradient elution	–	UV–VIS	Interna standard	Svasti et al. (2001); Vatanavicharn et al. (2012)
Plasma	His, Phe, Trp	ODS-3 (5 µm, 250 × 4.6 mm)	40 mM sodium perchlorate and 40 mM phosphate buffer, pH 3.5:acetonitrile (75: 25, v/v), isocratic elution	1	UV–VIS, 210 nm	Trp, Phe, His: 5, 10, 30 µg/L, respectively	Rezazadeh et al. (2013)
	Ile, Leu, Val	The pillar array column in a microchip (20 × 20 mm^2^ × 110 mm)	Water:acetonitrile:trifluoroacetic acid (92:8:0.02, v/v/v)	0.002	Fluorescence microscopy	Internal standard, LOD: 0.123, 0.130, 0.107 μM for Ile, Leu and Val, respectively	Song et al. (2013)
	Ala, Gly, Leu, Met, Orn, Phe, Tyr, Val	AAA RP18 (5 µm, 150 × 4.6 mm, 25 °C)	A water with 0.1%formic acid and 0.01% heptafluorobutyric acid, B acetonitrile with 0.1% formic acid and 0.01% heptafluorobutyric acid gradient elution	0.65	ESI–MS/MS	–	Johnson (2011)
	Ala, Arg, Asn, Asp, Cit, Cys, Gln, Glu, Gly, His, Ile, Leu, Lys, Met, Orn, Phe, Pro, Ser, Thr, Trp, Tyr, Val	SB-C1 8 (1.8 µm, 50 × 2.1 mm)	A: anionic ion-pair reagent, heptafluorobutyric acid solution in water, B: methanol, gradient elution	0.5	APCI-MS/MS	Calibration curve, LOQ 1 µM	Harder et al. (2011)
	Metabolites included: Ala, Arg, Asp, Cit, Cys, GABA, Gln, Glu, Gly, His, Ile, Leu, Lys, Met, Orn, Phe, Pro, Ser, Tau, Thr, Trp, Tyr, Val	Luna phenyl-hexyl	Acetonitrile, water and 0.1% acetic acid (pH 3.5–4.0)	–	TQMS	–	Lewis et al. (2008)
Serum	Phe, Trp, Tyr	C18 (5 µm, 250 × 4.6 mm, 50˚C)	Acetonitrile:water (1:9, v/v)	1	FLD	Calibration curve, LOD: 0.5, 0.014, and 0.0049 μM for Phe, Tyr and Trp, respectively	Li et al. (2011a, b)
	20 AA included: Ala, Arg, Asp, Cys, GABA, Glu, Gly, His, Ile, Leu, Lys, Met, Orn, Phe, Pro, Ser, Thr, Tyr, Val	Hypersil BDS C18 (5 µm, 200 × 4.6 mm, 35 °C)	A. acetonitrile:water (3:7, v/v) with 30 mM, pH3.7 ammonium/formic acid buffer), B. acetonitrile:water (5:5, v/v) with 30 mM, pH3.7 ammonium/formic acid buffer), C. acetonitrile:water (95:5, v/v), gradient elution	1	FLD, ESI MS/MS	Calibration curve, LOD 190–1170 µM	Li et al. (2011a, b)
	Ile, Leu, Lys, Met, Phe, Thr, Trp, Val	Luna (3 µm, 100 × 4.6 mm, 15 °C)	Acetonitrile (5 mM ammonium formate), gradient elution	0.6	ESI–MS/MS	Calibration curve, 0.05–750 ng	Calderón-Santiago et al. (2012)
	Ala, Arg, Asn, Asp, Cys, Gln, Glu, Gly, His, Ile, Leu, Lys, Met, Phe, Pro, Ser, Thr, Trp, Tyr, Val	C18 (5 µm, 150 × 4.6 mm, 15 °C)	Positive mode: 0.1% formic acid:acetonitrile (70:30 v/v) Negative mode: 0.1% ammonium hydroxide:acetonitrile (78:22 v/v)	0.8	ESI-QqQ	Internal standard	Chen et al. (2014)
Urine	44 AA included: Ala, Arg, Asn, Asp, Cit, Cys, GABA, Gln, Glu, Gly, His, Ile, Leu, Lys, Met, Orn, Phe, Pro, Ser, Tau, Thr, Trp, Tyr, Val	C18 (5 µm, 150 × 4.6 mm, 50 °C)	A: water, B: methanol + 0.1% formic acid + 0.01% heptafluorobutyric acids, gradient elution	0.8	API-MS/MS	Calibration curve, LOQ 1 µM for most AA	Held et al. (2011)
	44 AA included: Ala, Arg, Asn, Asp, Cit, GABA, Gln, Glu, Gly, His, Ile, Leu, Lys, Met, Orn, Phe, Pro, Ser, Tau, Thr, Trp, Tyr, Val	C18 (5 µm, 150 × 4.6 mm, 50 °C)	A: water + 0.1% formic acid + 0.01% heptafluorobutyric acids, B: acetonitrile, gradient elution	0.8	API-MS/MS	Internal standard, LOQ 0.5–50 µM	Kaspar et al. (2009)
	40 AA included: Ala, Arg, Asn, Asp, Cit, Cys, GABA, Gln, Glu, Gly, His, Ile, Leu, Lys, Met, Orn, Phe, Pro, Ser, Thr, Trp, Tyr, Val	AAA-MS column (250 × 2.0 mm, 25 °C),	A: 1 mM ammonium formate, B: 10 mM ammonium formate in methanol, gradient elution	0.25	ESI TQMS	Calibration curve, LOD ≤ 5 μM	Ubhi et al. (2013)
Serum, plasma and urine	Ala, Arg, Asn, Gln, His, Ile, Leu, Met, Phe, Ser, Thr, Trp, Tyr, Val	BEH-C18 (1.7 µm, 150 × 2.1 mm, 30 °C)	FLD: A. 20 mM sodium acetate buffer (pH 6.2), B. acetonitrile:methanol (7:93, v/v), gradient elution MS: exchanging sodium acetate with ammonium acetate	FLD: 0.35 MS: 0.2	FLD QqToF-MS	LOD 7.08–159.18 pmol/L	Müller et al. (2014)
Plasma, urine	52 AA included: Ala, Arg, Asn, Asp, Cit, Cys, GABA, Gln, Glu, Gly, His, Ile, Leu, Lys, Met, Orn, Phe, Pro, Ser, Tau, Thr, Trp, Tyr, Val	BEH C18 (1.7 µm, 100 × 2.1 mm, 30 °C)	A: 0.5 mM tridecafluoroheptanoic acid in water, B: 0.5 mM tridecafluoroheptanoic acid in acetonitrile, gradient elution	0.65	ESI MQTMS	LOQ ≤ 10 µmol/L	Waterval et al. (2009)
Plasma, urine and cerebrospinal fluid	26 AA included: Ala, Arg, Asn, Asp, Cit, Cys, Gln, Glu, Gly, His, Ile, Leu, Lys, Met, Orn, Phe, Pro, Ser, Tau, Thr, Trp, Tyr, Val	C18 (5 µm, 250 × 4.0 mm, 37 °C)	Acetate buffer (pH 5.25 or 6.8):acetonitrile, gradient elution	1 and 0.75	FLD	LOD ≥ 2 µmol/L	Sharma et al. (2014)
	33 AA included: Ala, Arg, Asn, Asp, Cit, Cys, Gln, Glu, Gly, His, Ile, Leu, Lys, Met, Orn, Phe, Pro, Ser, Tau, Thr, Trp, Tyr, Val	Column 1: porous graphitic carbon (PGC) (3 µm, 50 × 4.6 mm, 40 °C), Column 2: C18 (2.7 µm, 100 × 2.1 mm, 65 °C)	A: 1 mM tridecafluoroheptanoic acid in water, B. 1 mM tridecafluoroheptanoic acid in acetonitrile), gradient elution with back flush elution from column 1	0.3 or 0.35	ESI–MS/MS	Internal standard	Le et al. (2014)
Cerebrospinal fluid	Gly	MS: XDB-C18 (5 µm, 150 × 4.6 mm, 40 °C) FLD: C18 (5 µm, 60 × 4.0 mm, 30 °C)	MS: A: 0.1% formic acid in water, B: methanol, gradient elution FLD: A: 0.1 M sodium acetate buffer (pH 6.95):methanol:tetrahydrofuran (92.5:5:2.5, v/v/v), B: methanol:tetrahydrofuran (97.5:2.5, v/v), gradient elution	MS: 0.4 FLD: 1.2	ESI TQMS FLD	MS: LOQ 0.1 μM	Voehringer et al. (2013)

### Stationary phase and mobile phase

Reversed-phase (RP)-HPLC has become the most commonly used chromatography mode. In RP chromatography, the stationary phase is chemically modified to become non-polar (mostly by C8, C18). Typical stationary phases employed for RP-HPLC analysis of AAs include C18 and, more recently, C18s with reduced silanol activity (see Table2). With these new stationary phases, RP columns designed specifically for AA separation are more hydrophobic than classical C18 columns. Various C18 columns continue to be employed for the analysis of AAs as they provide good separation for a wide range of analytes. However, because many of the AAs are similar in structure, often with subtle differences in polarity, the use of the C18 column is often inadequate. Therefore, derivatization can be used (see sample preparation chapter). Additionally, as an option, a dedicated column for AA separation without derivatization (Intrada Amino Acid®) has appeared on the market. However, few publications have been found where authors use this column with orthogonal separation mechanism. Le and coworkers (Le et al. 2019) tested the separation of branched-chain amino acids (only valine, leucine and isoleucine) and trimethylamine-N-oxide on four columns including the Intrada Amino Acid column. However, their final choice was a C18-PFP column. This was due to the need to determine not only AAs but also the polar oxide. In recent years, there has been an increase in the number of publications using this new generation column, with some cases still using derivatization to increase the sensitivity of the method (Bala et al. 2021; Nomi et al. 2020; Watanabe et al. 2021).

Several different RP columns exist that vary in material, length, particle size, and internal diameter. In the analysis of biological samples, the use of columns with 1.7–3 µm particle size is acceptable. It should be noted that columns with a standard pore size (60 Å) may clog when the sample is insufficiently purified from the peptide. Therefore, it is preferable to use pre-columns or columns with larger pore size 200–300 Å.

For the mobile phase composition, mainly mixtures of organic solvents with water are used. In general, the separation takes place under gradient conditions. The composition of the phase depends on the choice of detection technique. For mass spectrometer detectors, it is preferable to add modifiers to the phase, such as tridecafluoroheptanoic acid (TDFHA, analysis with an ion pair), acetic acid or formic acid, as well as their sodium or ammonium salts. The details are listed in Table 2. This promotes ionization of the compounds, but also chromatographic peaks have a better shape (they are narrower and symmetrical), especially when the pH level is below the pKa of AAs (reversal of dissociation).

### Detection

The most common detection techniques connected with HPLC are UV or/and Vis absorbance, but not for the analysis of AAs. Diode-array detector (DAD) is an advanced UV–Vis detector commonly used to simultaneously and continuously acquire UV–Vis data with arrays of usually 512 photo-diodes. Unfortunately, these detectors could not be used for the determination of all AAs because most of these do not display absorbance on the required level in both the UV and Vis spectrum.

Generally, FLD is most often used for the determination of amino acids. It is based on the emission of photons from excited molecules having intrinsic fluorescence yields large enough to be detected analytically. Natively fluorescent compounds are relatively rare and include those containing at least two conjugated bonds or specific groups able to donate electrons to a system to enhance fluorescence intensity. In the case of determination of AAs, FLD can be used for compounds without natural fluorescence, provided chemical derivatization techniques have been used. These techniques, however, are cumbersome and are greatly influenced by many factors, e.g. mobile phase composition, temperature, reagent concentration, acidity. All this may significantly affect fluorescence intensity.

New analytical capabilities have enabled the introduction of mass spectrometers. Mass spectrometry (MS) and tandem mass spectrometry (MS/MS) are analytical techniques used to identify and quantitate compounds based on their molecular mass. This technique can help to provide confirmation of information of compounds using less specific detection methods such as DAD or FLD. MS is a very sensitive and specific technique that measures charged molecules. It involves sample vaporization followed by ionization, mass separation, and ultimately mass detection. The presence of the ion signal of each product within the sample is quantitatively detected, then plotted into mass spectra representing relative ion abundance as a function of the *m*/*z* ratio. MS/MS employs the use of two or more mass analyzers and is used to obtain additional information on ions generated by ionization. MS/MS is typically a two-stage mass analysis process separated by a step involving ion fragmentation. MS and MS/MS are excellent detection techniques for unambiguous AA identification. However, MS and MS/MS operations often incur high equipment cost and require highly trained personnel that often preclude its routine use in clinical evaluations. Due to the complexity of the matrix and to the ion suppression effect, sample preparation is necessary. The use of mass spectrometry is not preferred for all AAs, some of the compounds weakly ionize, so in such cases, it is preferable to use a fluorescence detector or derivatization to increase the ionization of the compounds. The matrix effect can be a problem for the analysis and should be checked (Calderón-Santiago et al. 2012; Waterval et al. 2009). This is equivalent to the purification or/and isolation of AAs from the sample. Harder and coworkers (Harder et al. 2011) proved that the phospholipids present in the samples affect the ion suppression of AAs. Unfortunately, this is time consuming and often requires the availability of reference material, but it is one of the steps of validation methods. It should be noted that retention time can shift when injecting different matrices (Müller et al. 2014). The advantage of using spectrometers is the possibility of using internal standards, which are AAs with substituted isotope atoms, not found naturally in the samples. Current availability of stable isotope-labeled standards is very wide (over 40 isotopes of AAs). The usage of labeled internal standards allows the assessment of the system reproducibility in every sample injection (Ubhi et al. 2013).

To increase the ionization of compounds, an ion-pairing agent can be used, for example, TDFHA. Methods using ion pair analysis are not universal. For TDFHA, Le and coworkers (Le et al. 2014) have concluded that the proposed analysis has good precision, sensitivity and reliability, particularly for polar compounds (glycine, phenylalanine, valine and methionine).

The unquestionable advantage of using liquid chromatography in connection with spectrometry to investigate AAs is that it reduces times of sample preparation and eliminates reagent-associated interferences and possible side reactions during derivatization. Additionally, a key advantage of HPLC is its separation power as it increases specificity and reduces the analytical complexity, which is especially important in case of MS. The isolation of the substance is extremely important to identify the compounds on the basis on parent and fragment anions, represented as chromatographic peaks of individual compounds. Unfortunately, this is associated with an increase in the limit of quantification, as for most MS detectors, limit of detection (LOD) is about 10 times higher than for FLD (see Table 2).

## Trends in developing HPLC analysis

Nowadays, the trends are often dispensed with automated techniques (based on ion chromatography) for reversed-phase or ion pair chromatographic systems (Held et al. 2011).

The development of AA analysis is associated with equipment miniaturization, the use of multidimensional separation and the introduction of new reagents or types of columns. Chromatographic miniaturization is combined with recent advances in high-resolution chromatography due to the use of special columns. It requires specialized pumps and equipment for high pressures up to 18.000 PSI because of the use of sub 2 µm particles in the stationary phase. This technique offers a number of distinct advantages including faster run times, narrower peaks, higher sensitivity, fewer sample and reagent requirements, better resolution and minimized matrix effects. Miniaturization also involves reducing the amount of sample needed for analysis. The use of microchips allows the analysis of biological samples available at the level of several microliters (Song et al. 2013).

Another advancement in liquid chromatography (LC) separation is a two-dimensional LC called 2D-LC or LC × LC. This methodology involves the coupling of two LC techniques thus providing increased peak capacity. A 2D-LC system typically includes the use of one or two HPLC systems, two pumps, and two columns, an injector, interface, and detector. The columns are connected by a transfer device that continuously collects effluent contents from the first column, then injects the contents in small, pre-defined amounts into the second column. For example, this solution is used by Le and co-workers (Le et al. 2014). The AAs are backward eluted off the first column and then separated on the second column.

There has also been development in the field of chromatographic columns, for which special types of packing or packaging methods are used, e.g. pillar array column, which are folded with low-dispersion turns. Such structures could effectively decrease eddy diffusion (Roux et al. 2011).

The analysis of AAs is a part of large-scale and long-term metabolomic studies. The repeatability is still an essential issue for the chromatographic-based methods. This subject needs attracted, widespread attention in the bionumerical studies and still remains challenging despite recent technique progresses (Luo et al. 2016; Cambiaghi and Ferrario 2017; Yang et al. 2021). For example, Yang et al. (2021) have constructed an online tool MMEASE enabling the integration of multiple analytical experiments. It enables integrating multiple analytical blocks and conducting enrichment analysis using many types of categories.

## Summary

Despite many years of amino acid analysis research, the isolation, separation and determination of amino acids from complex biological matrices continue to be a challenge. This is related to the use of many steps in the analytical methods, connected with extraction, separation, isolation and identification. The basic rules include the application of a minimal number of steps—each step means loss of precious material. Problems arise due to the surface of biological material (for example, blood, urine, serum) and the wrong choice of analytical procedures (each source demands individual purification strategy), and finally, the lower the amount of amino acid in the extract, the more purification factors are needed.

Alternatively, for HPLC, automated systems as well as gas chromatography can be used. (Kaspar et al. 2009). Kasper and co-workers compare these three techniques (GC–MS, LC–MS/MS and AA analyzer) regarding the use of deproteinization, minimum sample volume, minimum runtime, the number of amenable analytes, cost per analysis and limit of quantification (LOQ). Protein precipitation is not necessary only in the case of GC, but the number of amenable analytes is higher for LC. The cost per analysis for LC–MS/MS is similar as for the AA analyzer, but more expensive than GC. With regard to sample volume, minimum runtime and LOQ, LC is comparable to GC and both have much better parameters than AA analyzer.

The MS analyses are not entirely accurate with quantification. The obtained results may be affected by matrix effect that can lead to ionization suppression or enhancement, resulting in bias or reduced sensitivity of the method. The extent of matrix interference can vary considerably with the nature of the samples. As long as the required quantification limits can be achieved for the samples, samples can be diluted to minimize matrix effects. Unfortunately, in case of the MS detection derivatization may be needed to obtain the required sensitivity parameters (often < 1 µM). Accordingly, the use of the FLD is still most preferred.

The detection of rare and unfamiliar metabolic disorders requires, among others, AA analysis. There is a need for immediate follow-up based on research performed reliably. The use of mass spectrometers will be standard because the spectrum of compounds tested in one analysis is already huge. Time-consuming and high-cost techniques will be developed. Many scientists agree with this opinion (Lewis et al. 2008; Roux et al. 2011; Schulze et al. 2003).

## Data Availability

Not applicable.

## Abbreviations

2D-LC: Two-dimensional LC
AAs: Amino acids
APCI: Atmospheric pressure chemical ionization
API: Atmospheric pressure ionization
CSF: Cerebrospinal fluid
D-AAs: D-Amino acids
DAD: Diode-array detection
EDTA: Ethylenediaminetetraacetic acid
ESI: Electrospray ionization
FLD: Fluorescence detection
GC: Gas chromatography
HPLC: High-performance liquid chromatography
IBLC: Chiral thiol isobutyryl-l-cysteine
LC: Liquid chromatography
LCxLC: Two-dimensional LC
LOD: Limit of detection
LOQ: Limit of quantification
MCE: 2-Mercaptoethanol
MQTMS: Micromass quattro tandem mass spectrometer
MS: Mass spectrometry
MS/MS: Tandem mass spectrometry
OPA: Ortho-phthalaldehyde
QqToF-MS: Time of flight mass spectrometry
PEME: Pulsed electromembrane extraction
RP: Reversed phase
SPE: Solid-phase extraction
TDFHA: Tridecafluoroheptanoic acid
TQMS: Triple quadrupole mass spectrometer
UV: Ultraviolet
UV–Vis: Ultraviolet–visible spectrophotometric
Vis: Visible
